# Conversion Rates and Outcomes of Laparoscopic Appendectomy in Complicated Appendicitis: A Retrospective Study

**DOI:** 10.7759/cureus.90411

**Published:** 2025-08-18

**Authors:** FNU Sadaf, Muhammad Asif, Abdullah Iqbal, Paras Fatima, Amara Younas, Junaid Mushtaq, Izzah Shakeel

**Affiliations:** 1 General Surgery, Sadaf Khan Zada Clinic, Peshawar, PAK; 2 General Surgery, Shaheed Mohtarma Benazir Bhutto Medical University, Larkana, PAK; 3 General Surgery, Lahore General Hospital, Lahore, PAK; 4 General Surgery, Shalamar Hospital, Lahore, PAK; 5 General Surgery, Jinnah Hospital, Lahore, PAK; 6 General Surgery, Sir Ganga Ram Hospital, Lahore, PAK

**Keywords:** abcess, appendicitis, laparoscopic appendectomy, open appendectomy, perforation

## Abstract

Background

Laparoscopic appendectomy is a minimally invasive technique widely preferred for acute appendicitis due to less postoperative pain, faster recovery, and better cosmesis compared to open surgery. However, in complicated appendicitis, such as perforation, gangrene, abscess, or peritonitis-distorted anatomy, friable tissues, dense adhesions, and contamination can make the procedure technically demanding. These challenges may require conversion to open surgery, potentially increasing operative time, complications, and hospital stay.

Objective

To evaluate the conversion rate from laparoscopic to open appendectomy in patients with complicated appendicitis and to compare postoperative outcomes between converted and non-converted cases.

Methodology

This retrospective study included 393 patients who underwent laparoscopic appendectomy for complicated appendicitis at Shalamar Hospital, Lahore, Pakistan, from February 2022 to February 2025. Data were collected from patient records, operative notes, and discharge summaries. Variables included demographic details, intraoperative findings, reasons for conversion, operative time, postoperative complications, hospital stay, and 30-day readmissions. Statistical analysis was performed using SPSS version 25 (IBM Corp., Armonk, New York, USA), with a p-value < 0.05 considered significant.

Results

The conversion rate was 19.8% (n = 78). Common reasons for conversion included dense adhesions (41%), poor visualization (25.6%), and intraoperative bleeding (15.4%). Converted patients had significantly longer operative times (91.6 ± 22.4 vs. 61.7 ± 15.8 minutes, p < 0.001), higher surgical site infection rates (23.1% vs. 8.6%, p < 0.001), and longer hospital stays (5.6 ± 2.4 vs. 3.8 ± 1.6 days, p < 0.001). Risk factors associated with conversion included BMI > 30 (p = 0.004), CRP > 100 mg/L (p = 0.001), delayed presentation >48 hours (p = 0.001), and prior abdominal surgery (p = 0.03).

Conclusion

This study concludes that while laparoscopic appendectomy is effective for complicated appendicitis, conversion to open surgery is associated with increased morbidity. Early identification of high-risk patients based on clinical and laboratory parameters may help improve outcomes and surgical decision-making in complicated appendicitis.

## Introduction

Laparoscopic appendectomy has become widely accepted as the preferred surgical approach for acute appendicitis. It offers numerous advantages over open surgery, including reduced postoperative pain, shorter hospital stays, quicker return to normal activities, and better cosmetic outcomes [1]. Laparoscopic technique has repeatedly shown to give positive or similar results compared to the open approach in instances where the case of appendicitis is simple. Nevertheless, the issue is more complicated in the scenario of complex appendicitis, which encompasses the manifestations involving the perforation, gangrene, the abscess of an appendix, or generalized peritonitis [2]. There are considerable technical difficulties in complicated appendicitis because of fragmented anatomy, friable inflamed tissues, dense adhesions, and purulent or fecal contamination. These intraoperative challenges put one at risk of being converted to open surgery [3]. Conversion, described as the change of therapy taken during the operation, in which the laparoscopic procedure may not allow the continuity of the procedure, can be provocative in terms of patient safety. Despite the fact that the conversion must not be treated as a procedural failure, it is commonly linked to more prolonged operating conversion, higher intraoperative rates, along with postoperative morbidity, as well as longer length of hospital stays [4].

In the literature, there is a wide range of conversion rates in laparoscopic appendectomy in complicated appendicitis, with rates as low as five percent and as high as 25 percent. The difference is affected by the surgeon's experience, individual characteristics of the patient, resources available in the hospital, and the extent of intra-abdominal inflammation or contamination [5]. The literature also identifies that delayed presentation, inflammatory markers, increased body mass index, male gender, advanced age, and perforation/formation of the abscess on radiology are some of the clinical predictors of conversion in previous studies [6]. Identification of such risk factors before surgery can help prepare for the surgical process and have a more efficient perioperative care plan. The discussion about the best practice in complex appendicitis management is controverted [7]. Although some support initial non-operative management with the use of intravenous antibiotics with or without radiologically guided drainage, others support early laparoscopic intervention to provide source control. The combination of better surgical skills and laparoscopic devices has now enabled management of many complex cases that just a few years ago used to require open procedures [8].

However, in cases where visualization is inadequate or the risk of iatrogenic injury is high, prompt conversion to open surgery remains a prudent decision. It is also important to consider that worse outcomes in converted cases may reflect the severity of the disease rather than the act of conversion itself [9]. Studies have shown that patients who undergo conversion may experience higher rates of surgical site infection, intra-abdominal abscess, postoperative ileus, and longer hospital stays. Whether conversion independently influences outcomes or simply indicates a more severe clinical scenario is still a subject of investigation [10].

Objective

To evaluate the conversion rate from laparoscopic to open appendectomy in patients with complicated appendicitis and to compare postoperative outcomes between converted and non-converted cases.

## Materials and methods

Methodology

This retrospective cohort study was conducted at Shalamar Hospital, Lahore, Pakistan, from February 2022 to February 2025. The sample size was calculated using the formula:

\[n = \frac{Z^2 \cdot p(1 - p)}{d^2}\]

Where: Z = 1.96 for a 95% confidence interval, p = anticipated conversion rate in complicated appendicitis (assumed at 20% based on previous literature), d = precision or margin of error (set at 5%)\[ n = \frac{(1.96)^2 \cdot 0.20(1 - 0.20)}{(0.05)^2} = \frac{3.8416 \cdot 0.20 \cdot 0.80}{0.0025} \approx 246\]

To ensure greater validity and account for possible exclusions or incomplete records, a total of 393 patients were included using non-probability consecutive sampling.

Inclusion and exclusion criteria

Patients of any age and gender who were diagnosed intraoperatively with complicated appendicitis -- defined as perforation, gangrene, abscess, or generalized peritonitis -- and in whom a laparoscopic appendectomy was initially attempted were included in the study. Patients were excluded if they underwent a primary open appendectomy without an attempted laparoscopic approach, had an appendicular mass managed conservatively, underwent incidental appendectomy during unrelated surgical procedures, or had incomplete clinical, operative, or follow-up data.

Data collection

Data were collected retrospectively from patient files, operative notes, and discharge records using a structured proforma. Variables recorded included age, gender, duration of symptoms, intraoperative findings, conversion to open surgery, reason for conversion, operative time, postoperative complications, hospital stay, and readmissions within 30 days. The primary outcome was the conversion rate from laparoscopic to open appendectomy. Secondary outcomes included postoperative complications (surgical site infection, intra-abdominal abscess, ileus), operative duration, hospital stay length, and 30-day readmission rate.

Statistical analysis

Data were entered and analyzed using SPSS version 25 (IBM Corp., Armonk, New York, USA). Quantitative variables were reported as means ± standard deviation or medians with interquartile ranges. Categorical variables were expressed as frequencies and percentages. Associations between conversion and clinical variables were tested using the chi-square test for categorical variables and the independent t-test for continuous variables. A p-value < 0.05 was considered statistically significant.

## Results

Among 393 patients undergoing laparoscopic appendectomy, 78 (19.8%) were converted to open surgery. The mean age was higher in the converted group (36.9 ± 13.1 years) than in the non-converted group (32.6 ± 12.2 years). Males were more frequent among converted patients (53/78 (67.9%)) than non-converted (189/315 (60.0%)). Perforated appendix was seen in 44/78 (56.4%) of converted patients versus 124/315 (39.4%) in the non-converted. Abscess formation occurred in 30/78 (38.5%) vs. 74/315 (23.5%), respectively (Table 1).

**Table 1:** Demographic and clinical characteristics (n = 393). Data are represented as mean ± SD or percentages (%).

Variable	Total (n = 393)	Converted (n = 78)	Not converted (n = 315)
Mean age (years)	33.4 ± 12.6	36.9 ± 13.1	32.6 ± 12.2
Male, gender	242 (61.6%)	53 (67.9%)	189 (60.0%)
Duration of symptoms (days)	3 (1–7)	4 (2–7)	3 (1–6)
Perforated appendix	168 (42.7%)	44 (56.4%)	124 (39.4%)
Abscess formation	104 (26.5%)	30 (38.5%)	74 (23.5%)

Among the 78 converted cases, the most frequent indication was dense adhesions in 32 patients (41.0%). Poor visualization led to conversion in 20 (25.6%), intraoperative bleeding in 12 (15.4%), and iatrogenic injury in 6 (7.7%). Other causes accounted for 8 (10.3%) conversions (Table 2).

**Table 2:** Indications for conversion to open appendectomy (n = 78). Data are represented as percentages (%).

Reason for conversion	Frequency (%)
Dense adhesions	32 (41.0%)
Poor visualization	20 (25.6%)
Bleeding	12 (15.4%)
Iatrogenic injury	6 (7.7%)
Others	8 (10.3%)

Converted patients had a significantly longer operative time (91.6 ± 22.4 min) than non-converted (61.7 ± 15.8 min; p < 0.001). Surgical site infections were observed in 18/78 (23.1%) of converted vs. 27/315 (8.6%) of non-converted patients (p < 0.001). Intra-abdominal abscesses were more common in the converted group (7/78 (9.0%) vs. 10/315 (3.2%); p = 0.03), as was postoperative ileus (6/78 (7.7%) vs. 8/315 (2.5%); p = 0.04). Hospital stay was also longer for converted patients (5.6 ± 2.4 days vs. 3.8 ± 1.6 days; p < 0.001) (Table 3).

**Table 3:** Comparison of postoperative outcomes. Data are represented as mean ± SD or percentages (%). Significance level: p < 0.05.

Outcome	Converted (n = 78)	Not converted (n = 315)	t-value	χ²-value	p-value
Operative time (min)	91.6 ± 22.4	61.7 ± 15.8	11.71	–	<0.001
Surgical site infection	18 (23.1%)	27 (8.6%)	–	12.35	<0.001
Intra-abdominal abscess	7 (9.0%)	10 (3.2%)	–	4.76	0.03
Postoperative ileus	6 (7.7%)	8 (2.5%)	–	4.16	0.04
Length of hospital stay (days)	5.6 ± 2.4	3.8 ± 1.6	6.64	–	<0.001

Conversion was more common in patients with higher preoperative risk factors. BMI >30 was present in 19/78 (24.4%) of converted vs. 36/315 (11.4%) of non-converted patients (p = 0.004). Elevated WBC >14,000/mm³ occurred in 50/78 (64.1%) vs. 156/315 (49.5%) (p = 0.020). High CRP >100 mg/L was noted in 42/78 (53.8%) vs. 104/315 (33.0%) (p = 0.001). Delayed presentation (>48 h) was significantly associated with conversion (34/78 (43.6%) vs. 75/315 (23.8%); p = 0.001). Previous abdominal surgery was more frequent among converted cases (12/78 (15.4%) vs. 24/315 (7.6%); p = 0.030) (Table 4).

**Table 4:** Preoperative risk factors and their association with conversion. Data are represented as percentages (%). Significance level: p < 0.05.

Risk factor	Converted (n = 78)	Not converted (n = 315)	U-value	p-value
BMI > 30	19 (24.4%)	36 (11.4%)	8894.5	0.004
WBC > 14,000/mm³	50 (64.1%)	156 (49.5%)	9350.0	0.020
CRP > 100 mg/L	42 (53.8%)	104 (33.0%)	8148.5	0.001
Delayed presentation (>48 h)	34 (43.6%)	75 (23.8%)	8266.0	0.001
Previous abdominal surgery	12 (15.4%)	24 (7.6%)	9301.0	0.030

Readmission within 30 days occurred in 9/78 (11.5%) of converted vs. 12/315 (3.8%) of non-converted patients. Reoperation was needed in 5/78 (6.4%) converted cases and 4/315 (1.3%) non-converted cases. Emergency department visits without admission were more frequent among converted patients (11/78 (14.1%) vs. 20/315 (6.3%)). Overall, the total complication rate in the converted group was significantly higher at 26/78 (33.3%) compared to 45/315 (14.3%) (Table 5).

**Table 5:** Readmission and reoperation rates. Data are represented as percentages (%).

Outcome	Converted (n = 78)	Not converted (n = 315)
30-day Readmission	9 (11.5%)	12 (3.8%)
Reoperation required	5 (6.4%)	4 (1.3%)
Emergency room visit only	11 (14.1%)	20 (6.3%)
Total complication rate	26 (33.3%)	45 (14.3%)

## Discussion

This study evaluated the conversion rate and clinical outcomes associated with laparoscopic appendectomy in complicated appendicitis. Overall conversion rate of 19.8 percent was recorded, which falls within the range reported in the literature so far, with conversion rates differing between five percent and 25 percent based on the severity of disease, expertise of surgeons, and resources available in the institution. The results highlight that, despite the possibility of laparoscopic surgery in the vast majority of the complex ones, a large percentage of them still have a problem of the necessity to convert to an open procedure owing to technical and anatomical difficulties. The main causes of conversion, as presented in this group, are adhesions, difficulty visualizing, and intraoperative hemorrhage and agree with other reports that noted these factors as some of the major obstacles in the laparoscopic procedure completion [11]. Converted patients were more likely than non-converted patients to show delayed presentation, more severe inflammatory markers, and findings such as perforation and abscess, indicating that a higher frequency of delayed diagnosis and advanced disease stages contributes to surgical complexity [12]. Compared to the converted group, postoperative results were significantly poorer. Such patients experienced more procedure time, surgical site infection, and intra-abdominal abscess, along with increased hospital stay. The results comply with other studies indicating that conversion is possibly correlated with morbidity [13]. Nonetheless, one should take into account that conversion per se might not produce adverse effects directly, but represents a proxy of the severity of the disease. The more advanced pathology is probably the reason behind the higher complication rates among converted patients rather than conversion itself. High BMI, leukocytosis, high CRP levels, late presentation, and previous abdominal surgery were preoperative risk factors significantly related with an increased conversion rates [14]. This finding can be helpful clinically in that it can help to determine the high-risk patients in advance. The risk of conversion in these cases could be anticipated, and this would assist in selling surgical plans, counseling the families, and ensuring adequate readiness of resources, particularly in low-resource settings [15].

It is quite notable that more than two-thirds of patients who sought conversion appeared more than 48 hours after the start of the symptoms. This gives significance to early diagnosis and referral, especially in low- and middle-income countries where late access to care is becoming prevalent [16]. By intervening earlier, the development of the disease can be minimized, and the necessity of conversion will be moderate, resulting in better patient outcomes. Reoperation and readmission were also greater in the converted group, thus further supporting the relationship between conversion and poor postoperative outcomes [17]. These results indicate that tighter follow-ups of such patients deserve consideration and maybe the establishment of special postoperative care regimens for those at risk.

This study had several limitations. Being retrospective in nature, it was subject to selection and documentation bias, relying on the completeness and accuracy of patient records. As a single-center analysis, the findings may not be generalizable to other institutions with different surgical expertise, patient populations, or resource availability. Surgeon experience and intraoperative judgment, which can influence conversion decisions and outcomes, were not standardized or analyzed separately. Additionally, long-term postoperative outcomes beyond 30 days, such as incisional hernias or chronic pain, were not evaluated. Imaging findings and intraoperative grading of disease severity were not consistently available across all cases, limiting further stratification of risk. Despite these limitations, the study offers meaningful insights into the predictors and consequences of conversion in laparoscopic appendectomy for complicated appendicitis in a tertiary care setting.

## Conclusions

It is concluded that laparoscopic appendectomy is a safe and feasible approach for managing complicated appendicitis; however, a notable proportion of cases may require conversion to open surgery. The conversion rate in this study was 19.8%, with dense adhesions, poor visualization, and intraoperative bleeding being the most common causes. Conversion was significantly associated with longer operative times, higher postoperative complication rates, prolonged hospital stays, and increased readmission and reoperation rates. Preoperative factors such as elevated CRP, high BMI, delayed presentation, and prior abdominal surgery were predictive of conversion.
